# Analysis of the Urine with Special Reference to the Diseases of the Genito Urinary Organs

**Published:** 1879-11

**Authors:** 


ANALYSIS OF THE URINE WITH SPECIAL REFERENCE TO THE DISEASES OF THE GENITO URINARY ORGANS. By K. B. Hofman, Prof. in the University of Gratz, and R. Ultzman, Docent in the University of Vienna. Translated by T. Barton Brune, A.M., M.D., Resident Physician Maryland University Hospital, and H. Holbrook Curtis, Ph. B. D. Appleton & Co., N. Y. 1879.

We have received a copy of this same work previously, translated by Dr. F. F. Forcheimer, and published by Peter G. Thompson, Cincinnati. Upon comparison of the two we cannot discover any special superiority of either, but are ready to say that they are the best hand books of examination of the urine that we have noticed.

